# Supplementary material for: High precision epidermal radio frequency antenna via nanofiber network for wireless stretchable multifunction electronics
Source: Nat Commun. 2020 Nov 6;11:5629. doi: 10.1038/s41467-020-19367-8 (PMC7648760; doi:10.1038/s41467-020-19367-8)
Supplement: Supplementary file 1 — Supplementary Information [file 41467_2020_19367_MOESM1_ESM.pdf]

Supplementary Information

High precision epidermal radio frequency antenna *via* nanofiber network for wireless stretchable multifunction electronics

Zhang et al.

Table of Contents

Supplementary Figure..1

Supplementary Table..16

Supplementary Notes..20

Supplementary Note 1: Microstructure of Ag NFs under tensile strain........................ ***20***

Supplementary Note 2: Analysis of Skin Effect of the Ag NFs electrode...................... ***22***

Supplementary Note 3: Theoretical calculation of Ag NFs electrodes inductance....... ***24***

Supplementary Note 4: Inductance model and one-port scattering analysis................ ***28***

Supplementary Note 5: Finite element simulation of the mechanical property............ ***30***

Supplementary Note 6: Transmission model and two-port scattering analysis............. ***33***

Supplementary Note 7: Applications in functional wireless electronics........................ ***36***

Supplementary Methods..39

Preparation process of the Ag NFs electrode... ***39***

Fabrication process of the integrated circuit... ***39***

Different patterns used in the experiments.. ***40***

Finite element method (FEM) modelling... ***40***

Supplementary References..41

Supplementary Figure

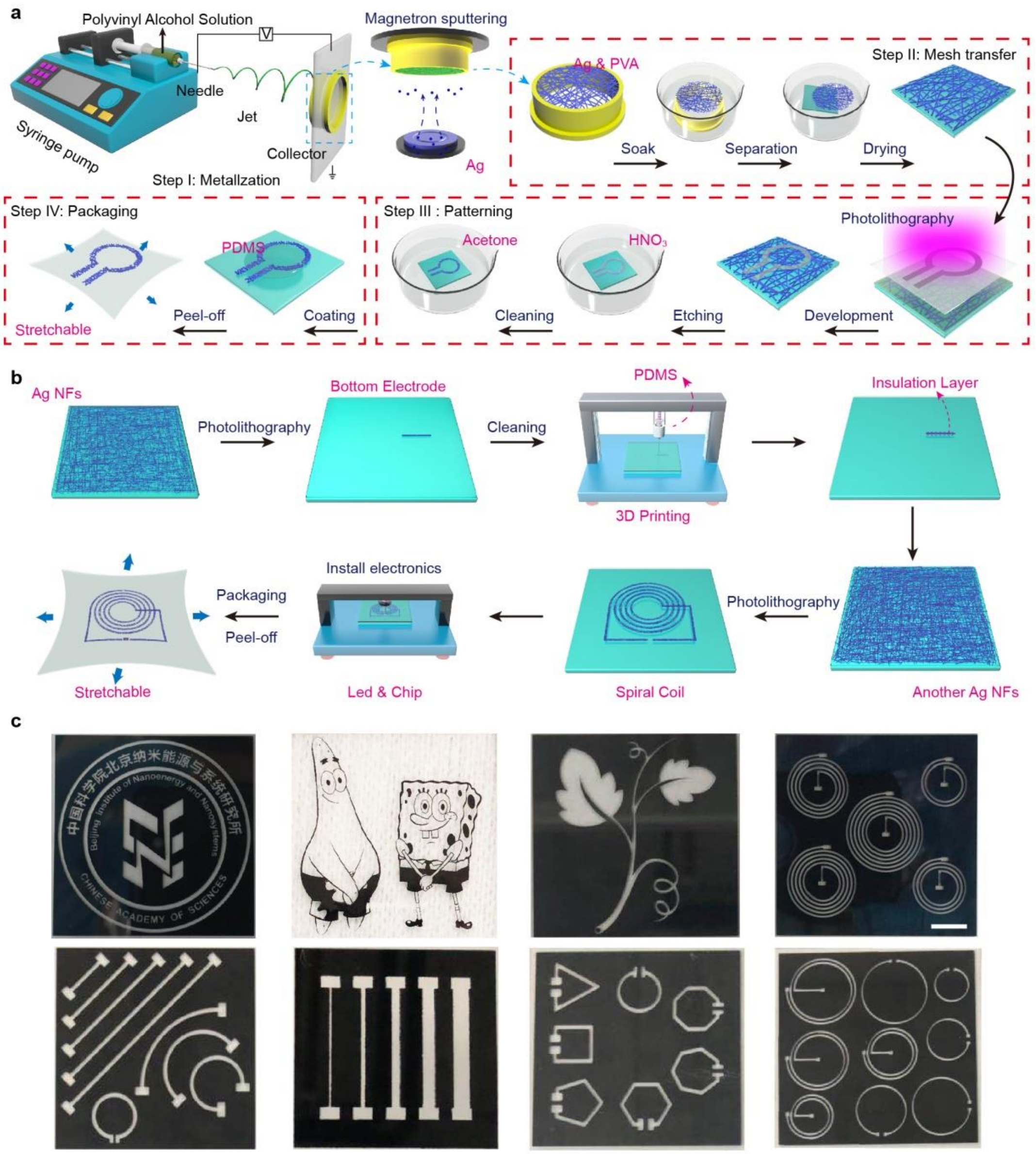

Supplementary Figure 1 | Fabrication process of stretchable transparent devices. (**a**) Schematic of preparation process for single-turn patterned Ag NF coil. (**b**) Schematic fabrication diagram of complex multi-functional integrated circuit. (**c**) Different patterns used in the experiment. (scale bar: 1 cm).

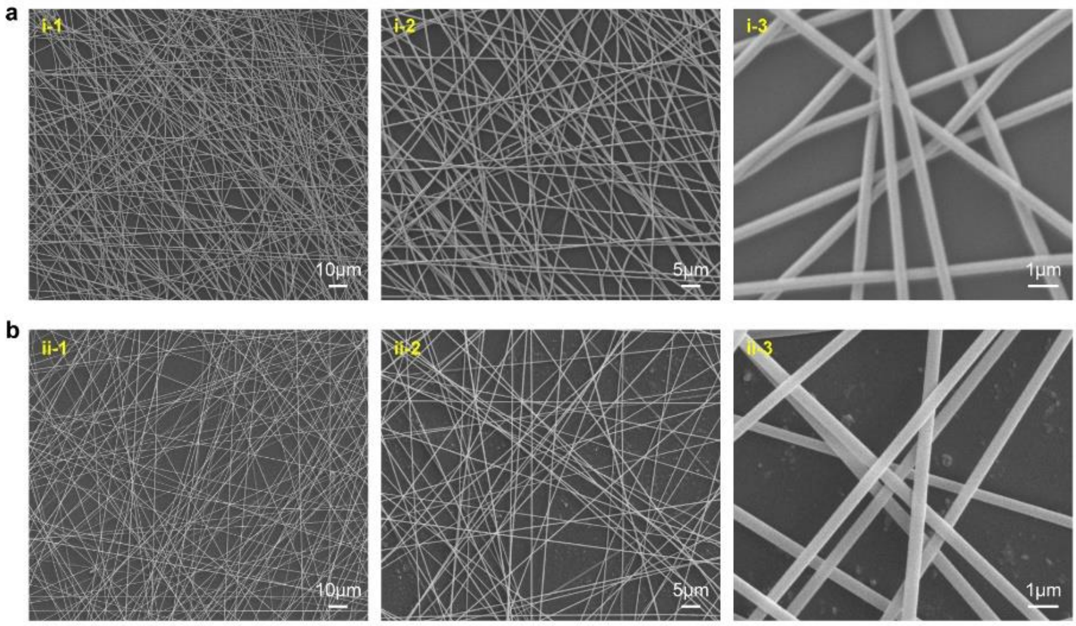

Supplementary Figure 2 | SEM images of Ag NFs electrodes in the original state without tensile strain. (**a**) SEM images of Ag NFs (sample 1) under different magnifications. (**b**) SEM images of Ag NFs (sample 2) under different magnifications.

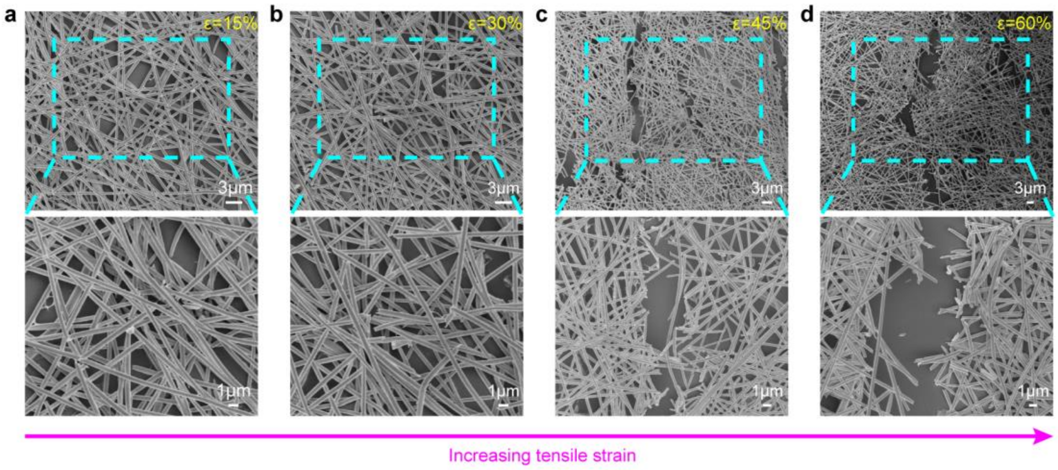

Supplementary Figure 3 | SEM images of Ag NFs under different tensile strain (without PDMS packaging). Four samples with the same processing conditions. (**a-d**) SEM images of Ag NFs under 15% strain (**a**, sample 1), 30% strain (**b**, sample 2), 45% strain (**c**, sample 3) and 60% strain (**d**, sample 4) at different magnifications.

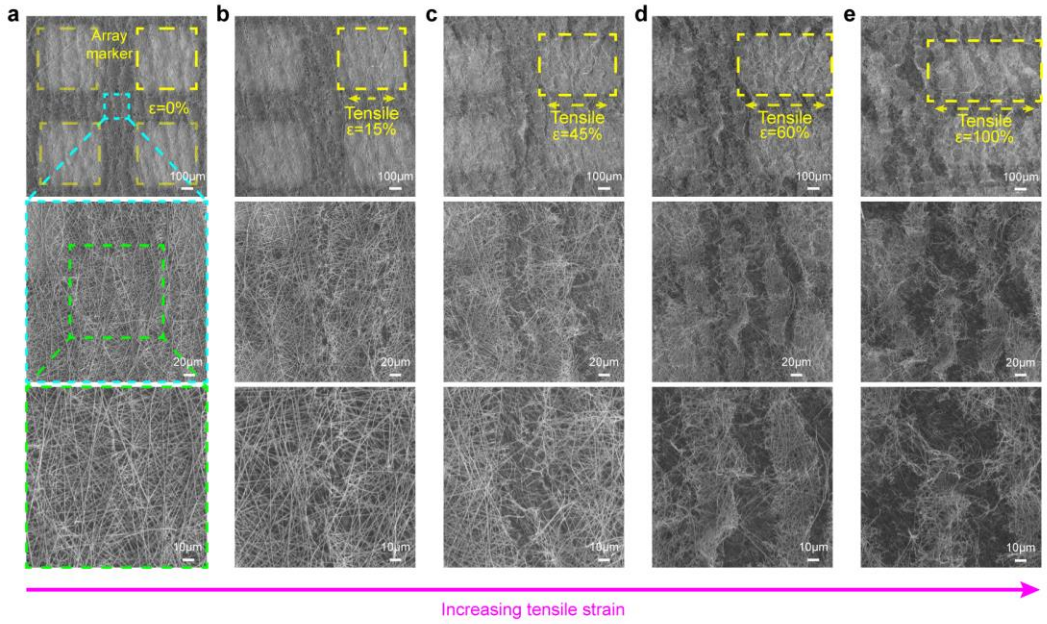

Supplementary Figure 4 | SEM images of Ag NFs under different tensile strain (without PDMS packaging). All SEM images are taken at the same place. (**a-e**) SEM images of Ag NFs under 0% strain (**a**), 15% strain (**b**), 45% strain (**c**), 60% strain (**d**) and 100% strain (**e**) at different magnifications.

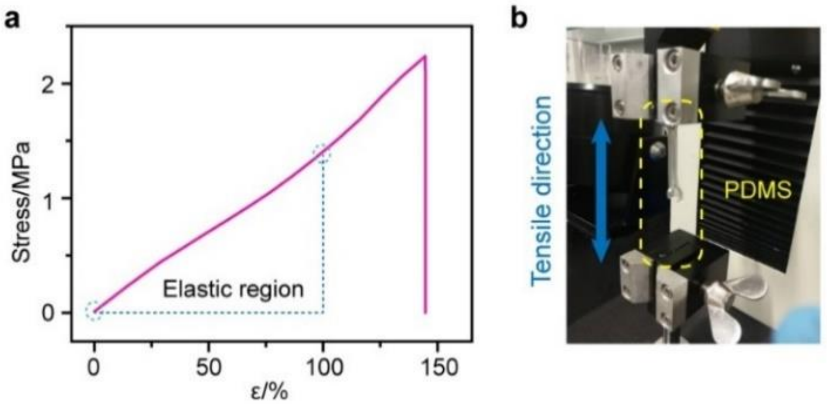

Supplementary Figure 5 | One-way drawing of the PDMS substrate. (**a**) Uniaxial tensile test of the PDMS substrate. (**b**) Schematic diagram of the measurement device.

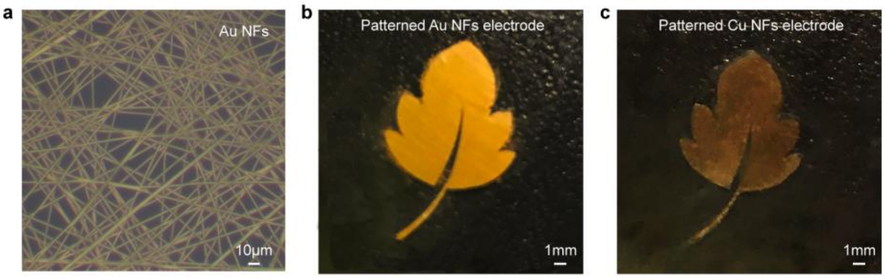

Supplementary Figure 6 | Various patterned electrodes of nanofibers. (**a**) Optical image of the Au NFs. (**b-c**) Patterned Au NFs (**b**) and Cu NFs (**c**) electrode.

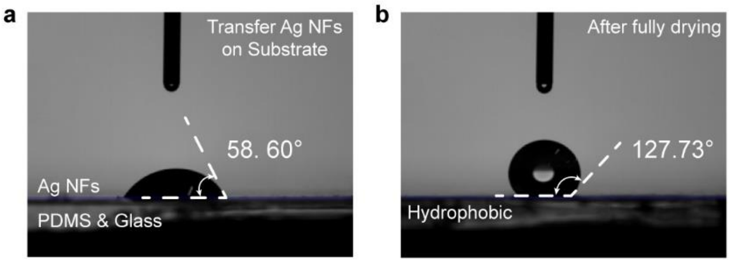

Supplementary Figure 7 | Contact angle of Ag NFs on the PDMS substrate. (**a**) Contact angle of Ag NFs after transferring it on the PDMS substrate. (**b**) Contact angle of Ag NFs after fully drying.

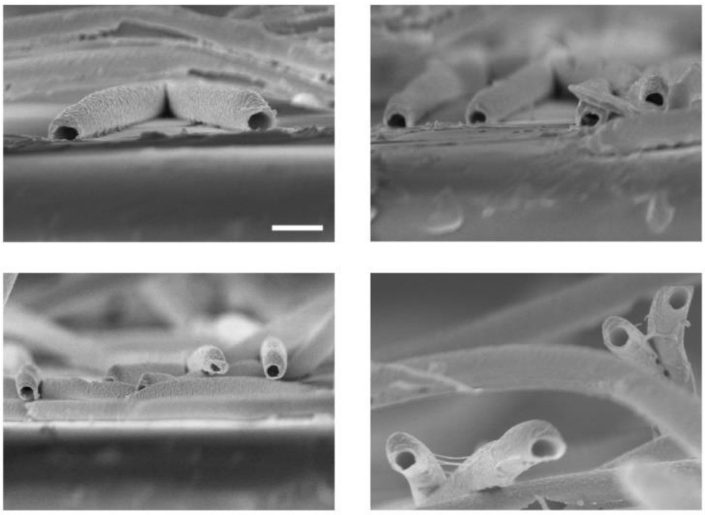

Supplementary Figure 8 | Cross-sectional SEM images of the core/shell Ag NFs (scale bar: 1 μm).

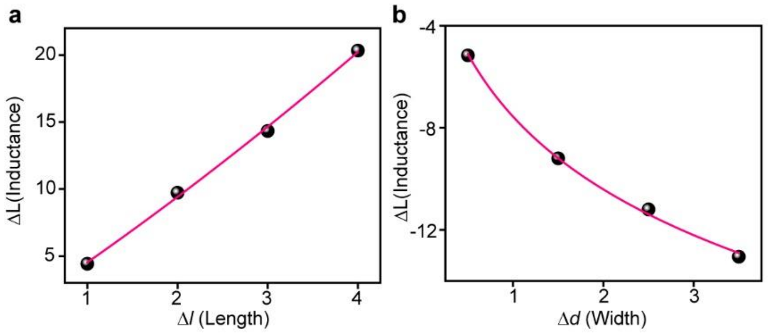

Supplementary Figure 9 | Fitting of the straight electrode with inductance variation versus the increment in length (a) and width (b).

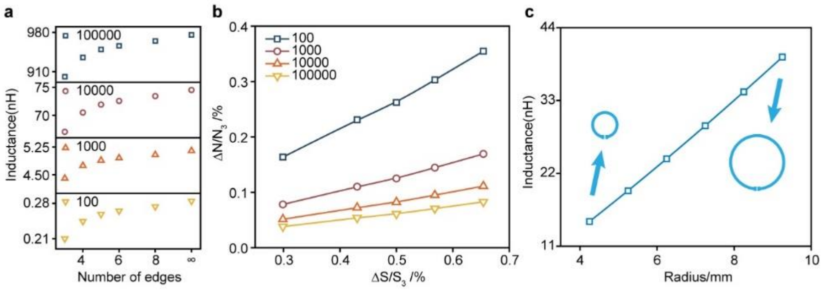

Supplementary Figure 10 | Theoretical inductance calculation of various shape electrodes. (**a**) Inductance of Ag NFs electrodes with different numbers of edges. (**b**) Comparison of inductance changes of different electrode lengths with the same area variation. (**c**) Inductance variation of circular electrodes with different radii.

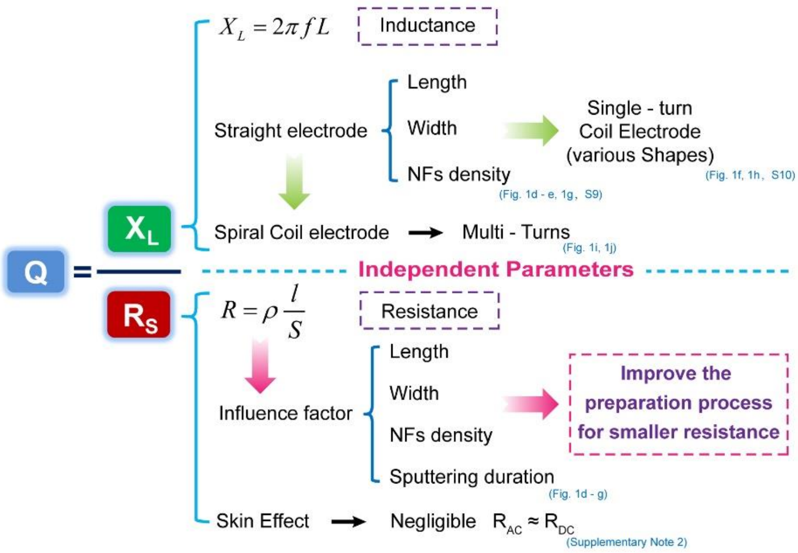

Supplementary Figure 11 | Research approach and the article logic structure.

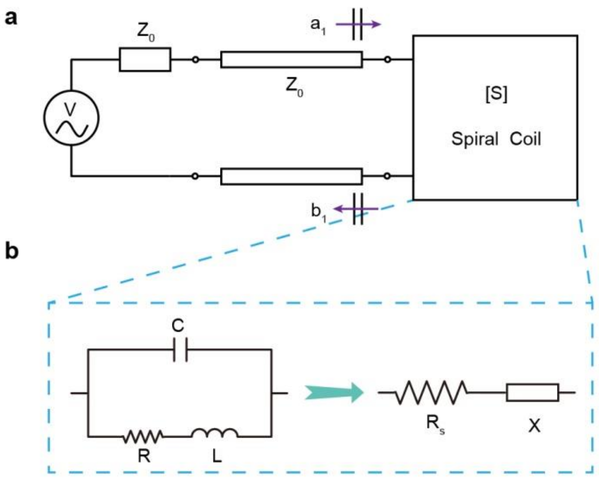

Supplementary Figure 12 | One-port scattering analysis method and equivalent inductance model. (**a**) Schematic diagram of Ag NFs coil using vector network analyzer for one-port microwave scattering experiment. (**b**) Lumped-parameter equivalent circuit of Ag NFs coil and its equivalent series circuit.

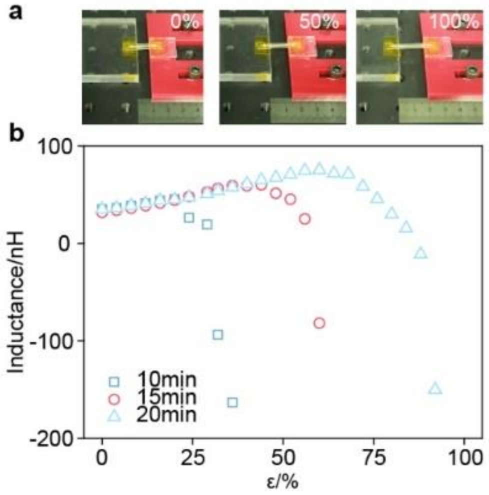

Supplementary Figure 13 | Straight Ag NFs electrode inductance variation versus tensile strain. (a) Digital photos of straight electrode under various strains. **(b)** Inductance variation of straight electrode under different strains with three electrospinning durations.

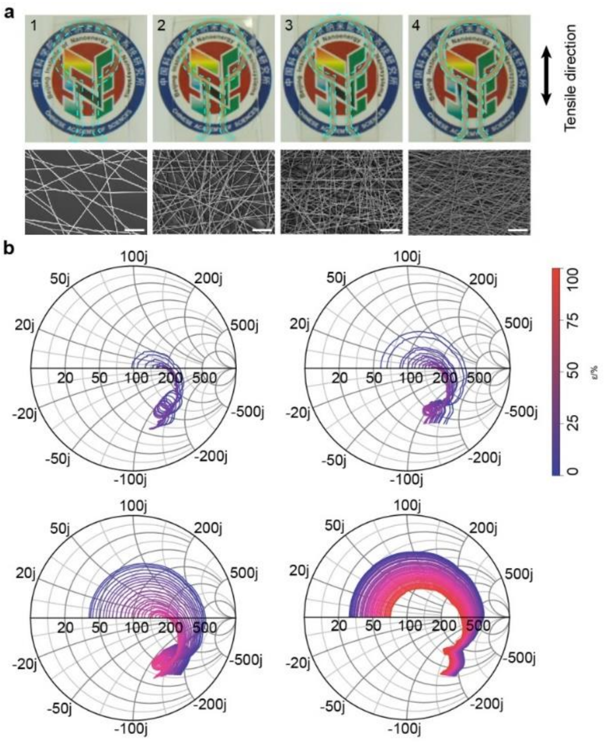

Supplementary Figure 14 | Single-turn Ag NFs coil with different densities versus the tensile strain. (**a**) Digital photos of single-turn Ag NFs coil and the corresponding SEM images with four electrospinning durations (scale bar: 50 μm). (**b**) Smith chart of single-turn Ag NFs coil under tensile strain.

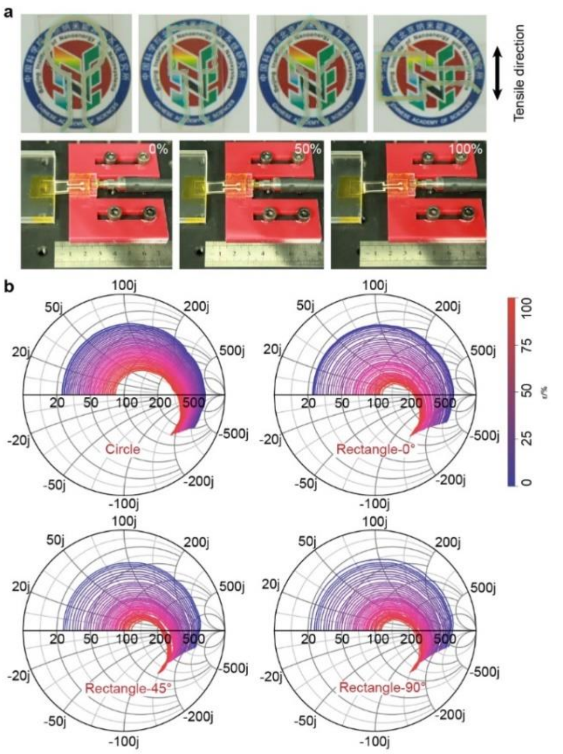

Supplementary Figure 15 | Single-turn Ag NFs coil with different shapes versus tensile strain. (**a**) Digital photos of single-turn Ag NFs coil with different shapes and rectangle coil under various tensile directions. (**b**) Smith chart of single-turn Ag NFs coil under tensile strain.

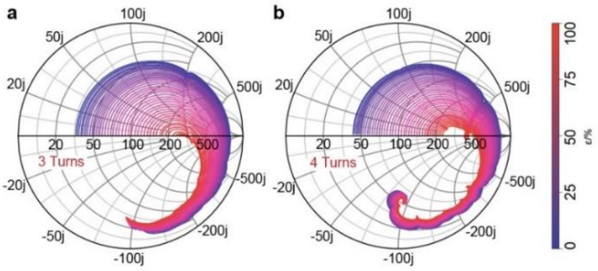

Supplementary Figure 16 | Smith chart of Ag NFs spiral coil versus tensile strain. (**a**) Three-turn Ag NFs spiral coil versus tensile strain. (**b**) Four-turn Ag NFs spiral coil versus tensile strain.

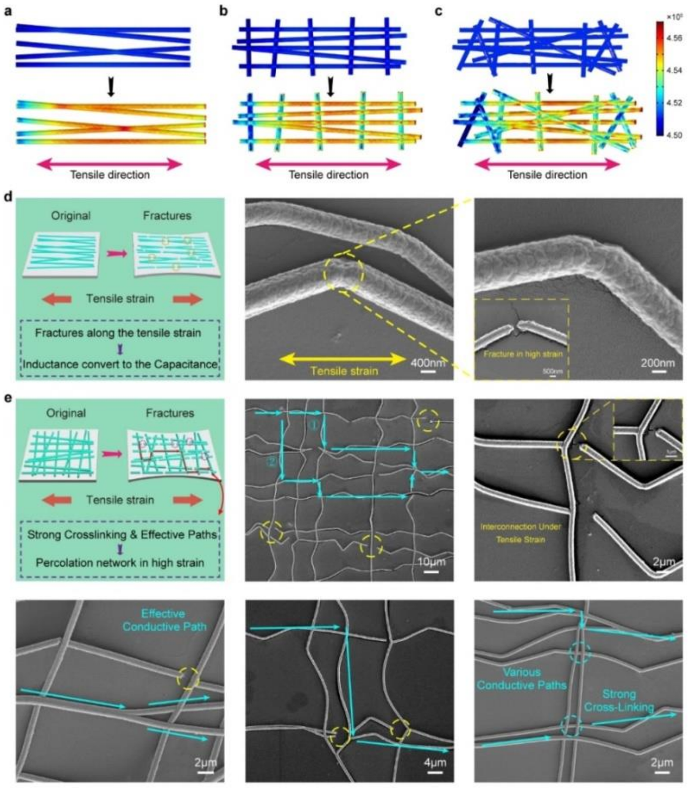

Supplementary Figure 17 | Simulation and SEM images of the Ag NFs with tensile strain. (**a-c**) Simulations of the stress distribution of unidirectional Ag NFs (**a**), bidirectional Ag NFs (**b**), and random orientation Ag NFs (**c**) under tensile strain. (**d-e**) Schematic diagram and SEM images of the evolution of the unidirectional Ag NFs (**d**) and multi-orientation Ag NFs (**e**) under tensile strain.

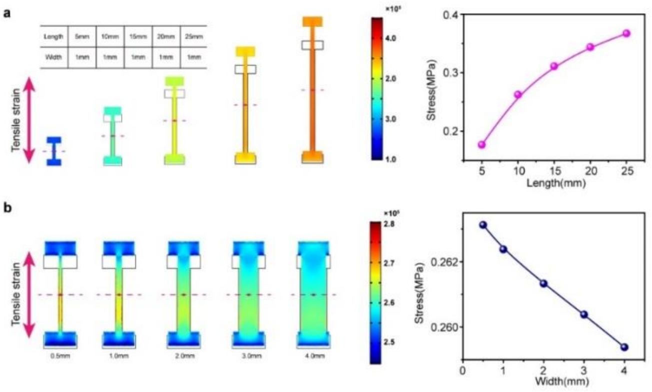

Supplementary Figure 18 | Finite element simulation of straight Ag NFs electrodes versus tensile strain. (**a**) Ag NFs electrodes with different lengths under tensile strain. (**b**) Ag NFs electrodes with different widths under tensile strain.

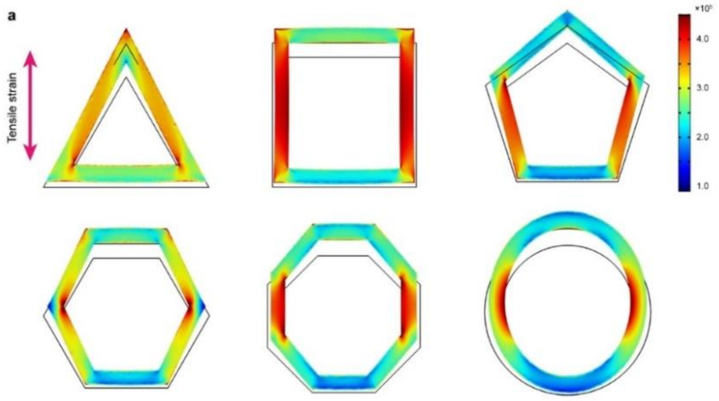

Supplementary Figure 19 | Finite element simulation of Ag NFs electrodes with different shapes versus tensile strain.

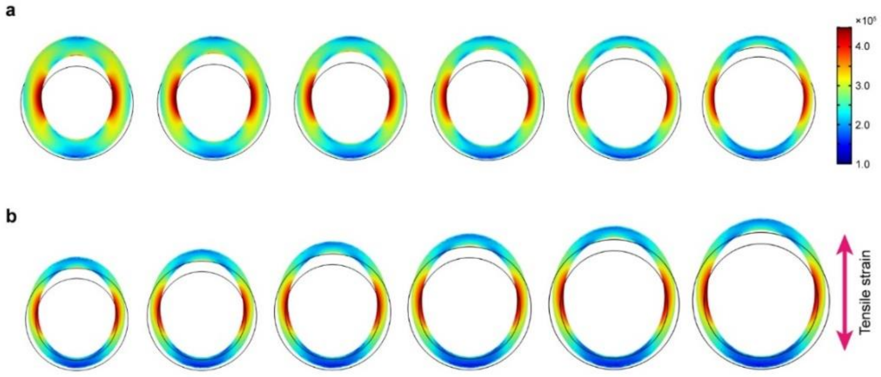

Supplementary Figure 20 | Finite element simulation of circular Ag NFs electrodes with different lengths (a) or widths (b) under tensile strain.

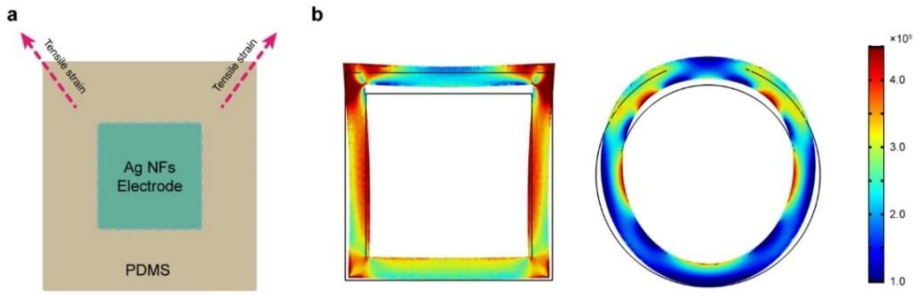

Supplementary Figure 21 | Finite element simulation of Ag NFs electrodes with different tensile direction. (**a**) Schematic diagram of stretching model in COMSOL. (**b**) Simulation results of the square and circular electrodes.

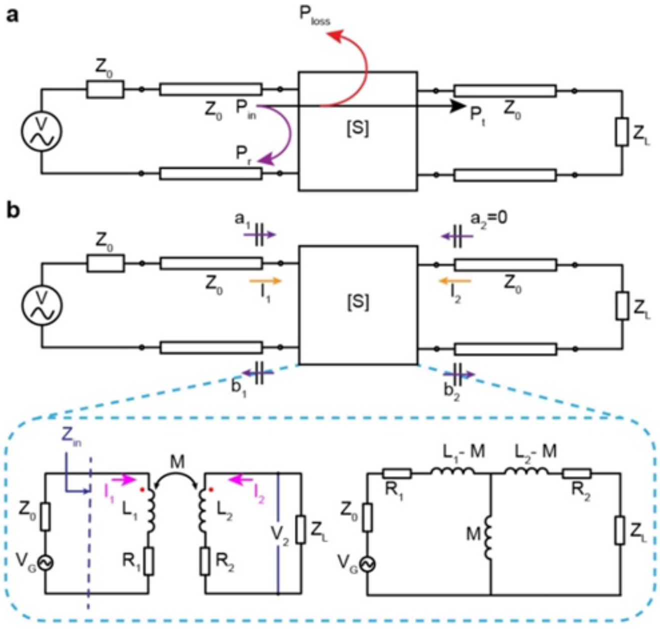

Supplementary Figure 22 | Two-port microwave scattering analysis and wireless transmission circuit.** (**a**) Schematic diagram of wireless power transfer based on the two-port microwave scattering experiment. (**b**) Fundamental wireless transmission circuit with a mutually coupled coil and equivalent *T* circuit.

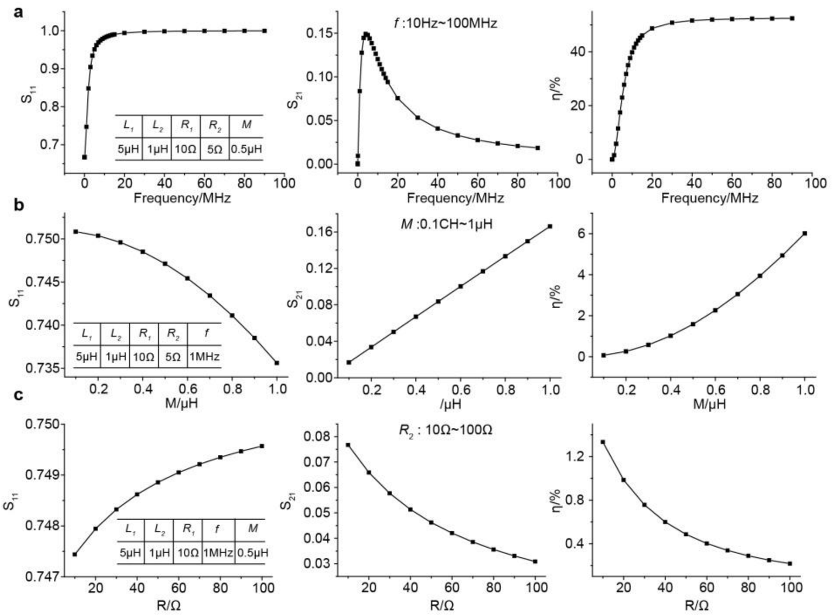

Supplementary Figure 23 | Theoretical calculation for the parameter affecting wireless transfer efficiency. (**a**) Effect of frequency (f) on S parameters and transfer

efficiency. (**b**) Effect of mutual inductance (*M*) on *S* parameters and transfer efficiency. (**c**) Effect of receiver resistance (R_2) on *S* parameters and transfer efficiency.

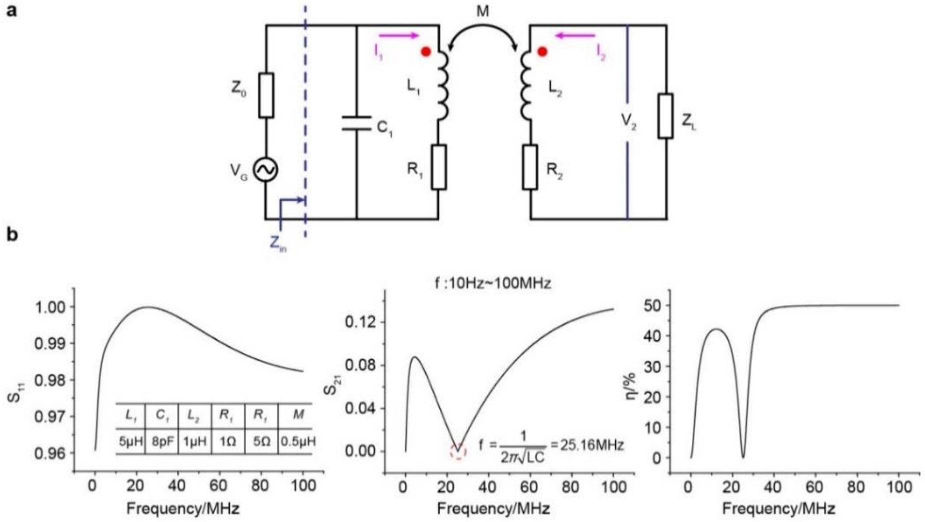

Supplementary Figure 24 | Theoretical calculation for mutually coupled coil with capacitance for wireless transfer efficiency. (**a**) Equivalent circuit of wireless power transfer. (**b**) Effect of frequency (*f*) on *S* parameters and transfer efficiency.

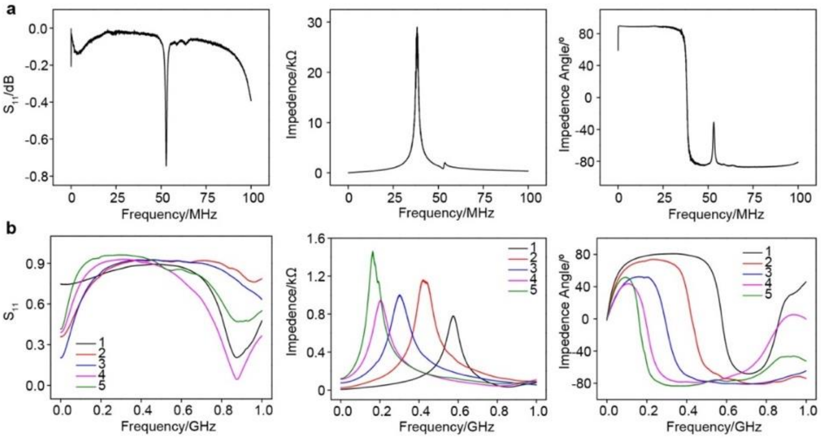

Supplementary Figure 25 | Characterization of primary coil and stretchable spiral coils. (**a**) S_{11}, impedance, and impedance angle of commercial transmission coil. (**b**) S_{11}, impedance, and impedance angle of stretchable spiral coils with various numbers of turns.

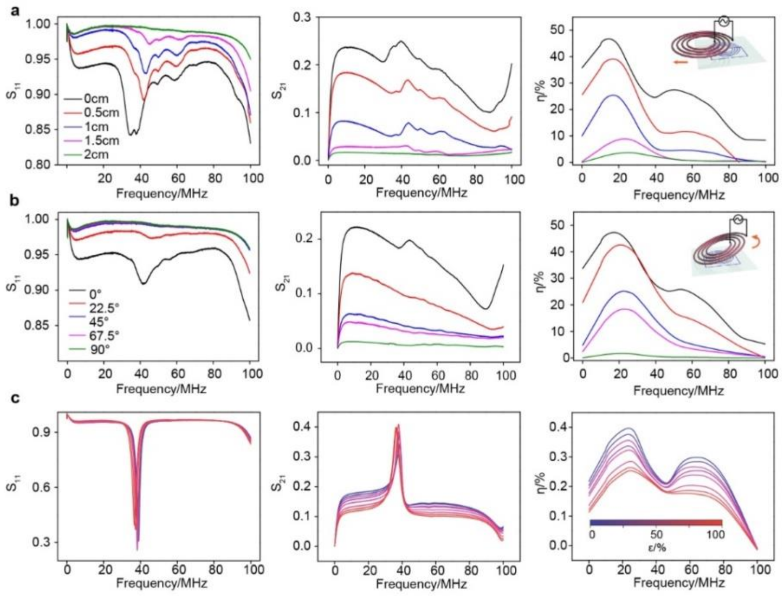

Supplementary Figure 26 | Characterization of the coupling between the primary coil and the stretchable spiral coil. (**a**) S_{11}, S_{21}, and transfer efficiency at different lateral distances between the primary coil and the stretchable coil. (**b**) S_{11}, S_{21}, and transfer efficiency at different rotation angles between the primary coil and the stretchable coil. (**c**) S_{11}, S_{21}, and transfer efficiency with the stretchable coil under tensile strain.

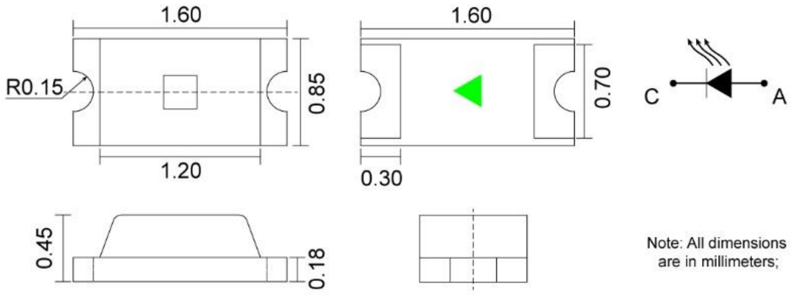

Supplementary Figure 27 | Specific dimensions of the chip-LED.

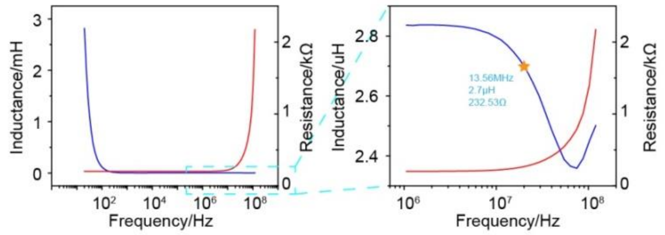

Supplementary Figure 28 | Characterization of NFC spiral coils (13.56 MHz, 2.7 μH)

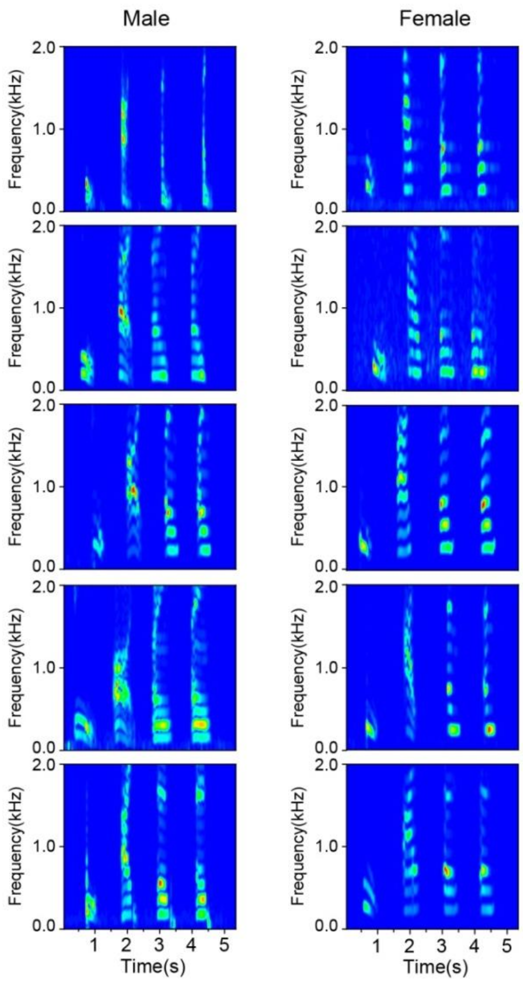

Supplementary Figure 29 | Audio spectrum of males and females ("B", "I", "N", and "N").

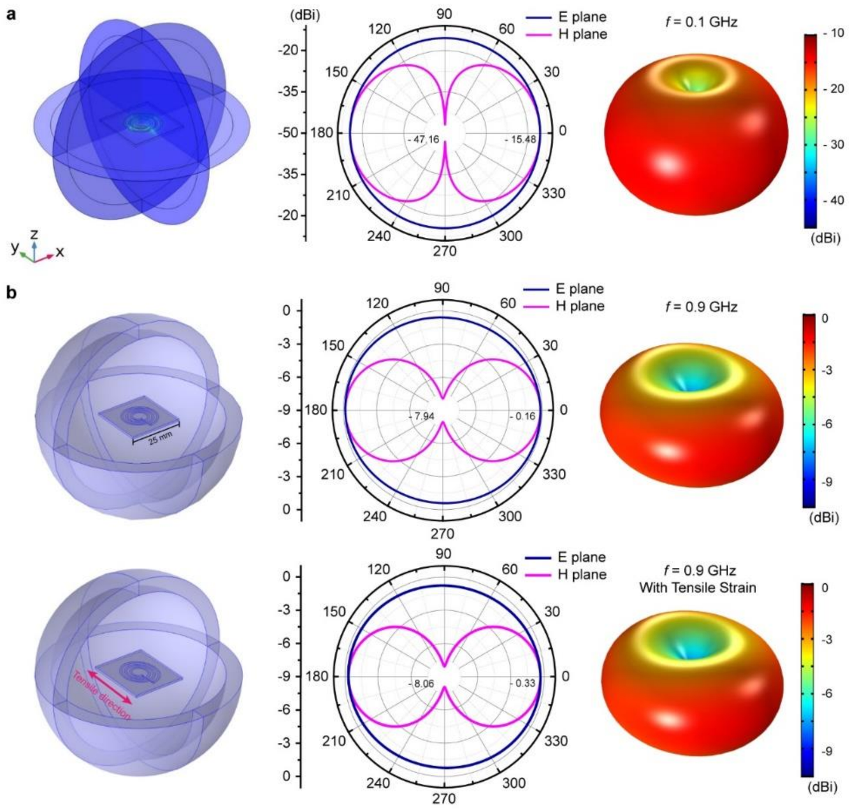

Supplementary Figure 30 | Finite element simulation of Ag NFs antenna gain radiation pattern. (**a**) Simulated Ag NFs antenna gain patterns at 0.1 GHz. (**b**) Simulated Ag NFs antenna gain patterns at 0.9 GHz.

Supplementary Table

Summary of the state-of-the-art several key works about the Ag NFs

Conventional Ag NWs electrodes: the conventional Ag NWs are grown by reducing the Ag NO_3 in the EG solution. The length of Ag NWs is 20 to 150 μm, and the diameter is 30 to 150 nm. The Ag NWs solution is uniformly distributed in the elastomer matrix, which could fully embrace the percolated conductive filler network and protect it from strain-induced fracture. Although the electrodes based on the conventional Ag NWs possess the excellent stretchability, more NWs junctions lead to a large resistance and it is difficult to make patterns by using photolithography. Some research works have been reported that the NWs junction could be improved by flash-induced electron excitations or depositing other materials on the interconnection, such as Au, Pt, CNTs, etc. Despite these attempts, none of the alternatives have succeeded in demonstrating high performance of all transparency, conductivity, stretchability and high precision patterned electrodes.

As-synthesized Ag NFs:

(1) PVA NFs prepared by electrospinning possess a high aspect ratio, and the length of nanofibers can reach up to several centimeters. This will improve the electrode tranparency and conductivity.

(2) Magnetron sputtering is a simple method for preparation of Ag NFs, which will deposit silver on the surface of PVA NFs. Noteworthily, Ag NFs are naturally interconnected at their junctions during metal deposition, which will make the Ag NFs possess the higher conductivity than conventional Ag NWs. The thickness and diameter of the Ag NFs could be controlled by sputtering time and electrospinning parameters, respectively. Moreover, the Ag NFs transfer process is carried out on the water surface, which can not only ensure the Ag NFs are flat on the PDMS substrate but also promote the dissolution of PVA. Meanwhile, the adhension force between Ag NFs and substrate becomes stronger owing to the Van der Waal's force after drying.

(3) The Ag NFs could be patterned by using the photolithography technique and wet etching, and the line width was exactly up to several tens of micrometers. The patterned Ag NFs will be packaged by liquid PDMS, which will help the electrodes with the excellent tensile properties. Furthermore, Ag NFs will form the core-shell structure after dissolving the PVA NFs which will weaken the skin effect to a certain extent, and it will be helpful for designing radio-frequency electronics.

Here, we have explored a set of mature technology for preparing various Ag NFs electrodes or radio frequency devices, and then do some comparsions for electrical and mechanical properties with other works. The results show that our as-synthesized Ag NFs electrodes exhibit the advantages of simple operation, low cost and easily large-scale preparation, which will provide a powerful platform for wearable electronics.

Supplementary Table 1 Summary of the state-of-the-art several works about the Ag NFs

Fabrication methods	Materials	Structure	Conductivity	Stretchability	Transmittance	Patterning accuracy	References
Electrospinning & Sputtering	Ag NFs/ PDMS	Core-shell Ag NFs embedded in PDMS	2.78×10^4 S cm^{-1} & 1.68 Ω sq^{-1}	100%	70%	20 μm	This work
Electrospinning & Homogeneous dispersion	Ag NWs/ (PU) NFs	Ag NWs randomly distributed PU NFs scaffold	9190 S cm^{-1}	310%	--	--	*Adv. Mater.* **2019**, 31, 1903446
Electrospinning & facile vacuum filtration	Ag NWs/ PA6 NFs	Ag NWs embedded into scaffold-reinforced conductive nanonetwork	8.2 Ω sq^{-1}	--	84.9%	--	*ACS Nano* **2018**, 12, 9326
Electrospinning & Sputtering	Ag NFs	Continuous nanotrough networks with concave structure	15 Ω sq^{-1}	50%	90%	--	*Nat. Nanotech.* **2013**, 8, 421
Electrospinning & Sputtering	Au namonesh	Au nanomesh attach on the skin	2.9×10^{-3} S	48%	--	500 μm	*Nat. Nanotech.* **2017**, 12, 907
Electrospinning & Sputtering	Au NFs/ PAN	Core-shell Au NFs on the PDMS	25 Ω sq^{-1}	80%	82%	--	*Adv. Mater.* **2013**, 3, 1332
Homogeneous dispersion	Ag NWs/ SBS	Ag NWs ($l \approx 30$ μm, d ≈ 150 nm)	1.2×10^4 S cm^{-1}	Conductivity maintained up to 100% strain	--	--	*ACS Nano* **2015**, 9, 6626
Homogeneous dispersion	Ag-Au NW/SBS	Core-shell Ag-Au NFs embedded in SBS	4.18×10^4 S cm^{-1}	266%	--	500 μm	*Nat. Nanotech.* **2018**, 13, 1048
Homogeneous dispersion & Screen Printing	Ag NW/methyl cellulose	Ag NWs ($l \approx 20$ μm, d ≈ 30 nm)	4.67×10^4 S cm^{-1}	70%	--	50 μm	*Adv. Mater.* **2016**, 28, 5986
Vacuum assisted filtration and transfer	Ag NW/PUA	Multiple times grown long Ag NWs ($l \approx 150$ μm), pre-strained eco-flex	9 Ω sq^{-1}	460%	--	--	*Adv. Mater.* **2012**, 24, 3326
Ag NW network soaked in GO dispersion	Ag NW-rGO/ PUA	GO reinforced Ag NFs junction connection	14 Ω sq^{-1}	140%	75%	--	*ACS Nano* **2014**, 8, 1590
Homogeneous dispersion	Ag NW/ polyacrylate	Ag NWs embedded in the surface layer of poly(acrylate) matrix	7.5 Ω sq^{-1}	50%	80%	--	*Nanotechnology* **2012**, 23, 344002
Homogeneous dispersion	Ag NW/PDMS	Partially embedded in elastomer (casting and peeling off)	2.64 Ω sq^{-1}	35%	62%	--	*J. Mater. Chem. C* **2014**, 2, 10369
Homogeneous dispersion	Ag NW/PDMS	Partially embedded in elastomer (casting and peeling off)	8130 S cm^{-1}	80%	--	--	*Adv. Mater.* **2012**, 24, 5117
Homogeneous dispersion	Ag NW/NIPAM	NIPAM polymerization on Ag NW aerogel	93 S cm^{-1}	800%	--	--	*Nat. Commun.* 2018, 9, 2786
Homogeneous dispersion	Ag NW-PEDOT:PSS/PU	Brush painting hybrid ink on PU substrate	19.7 Ω sq^{-1}	30%	88%	--	*Sci. Rep.* **2017**, 7, 14685
Homogeneous dispersion	Ag NW-PEDOT:PSS/ Diels-Alder elastomer	Lock Ag NW network with PEDOT	15 Ω sq^{-1}	100%	78%	--	*ACS Appl. Mater. Interfaces* **2015**, 7, 14140
Homogeneous dispersion	Ag NW-carbon nanofibers/PU	Ag NW-carbon nanofibers coated on the surface of PU foam	16.6 S cm^{-1}	140%	--	--	*Adv. Mater. Technol.* **2019**, 1900060
Homogeneous dispersion	Ag NWs	A periodic two-dimensional network	6.5 Ω sq^{-1}	--	91%	--	*Nano Lett.* **2012**, 12, 3138
Homogeneous dispersion	Ag NWs/ PVA	PVA solution spin-coated over Ag NWs network	70 Ω sq^{-1}	--	88%	--	*Adv. Mater.* **2010**, 22, 4484
Homogeneous dispersion	Ag NWs	Ag NWs ($l \approx 10$ μm) & coated Au on junction	20 Ω sq^{-1}	--	80%	--	*ACS Nano* **2010**, 4, 2955
Heterogeneous assembly	Ag NP/ SBS	Ag NP precursor absorbed in SBS fiber	5400 S cm^{-1}	100%	--	200 μm	*Nat. Nanotech.* **2012**, 7, 803
Homogeneous dispersion	Ag NWs	Long Ag NWs ($l \approx 150$ μm, d ≈ 100 nm)	15.6 Ω sq^{-1}	--	90%	--	*Nano-Micro Lett.* **2015**, 7, 51.
Homogeneous dispersion	Ag-Au core-shell NWs Pt black/ SBS	Free-standing composite film	11210 S cm^{-1}	50%	--	500 μm	*Adv. Mater. Technol.* **2019**, 00768
Direct printing	Ag NWs	Large-scale-aligned Ag NWs (d ≈ 695 nm)	26.9 Ω sq^{-1}	--	94.7%	--	*Adv. Mater.* **2016**, 28, 9109

Supplementary Table 2 Conductivity and permeability of PDMS and metals

	σ (S/m)	μ_r
PDMS	3.45×10^{-13}	1
Ag	5.00×10^{7}	0.999974
Au	4.88×10^{7}	0.999983
Pt	9.52×10^{6}	1.000260
Cu	5.88×10^{7}	0.999900
Cr	7.92×10^{3}	1.000140
Ni	1.44×10^{4}	120

Supplementary Table 3 Skin depth at different frequencies

f (Hz)	5 G	1 G	100 M	10 M	1 M
δ (μm)	1.066	2.251	7.118	22.508	71.177

Supplementary Table 4 Parameters of regular polygon electrode

n	3	4	5	6	8
$f(n)$	-1.40546	-0.77401	-0.40914	-0.16152	0.21198

Supplementary Table 5 Parameters of regular polygon electrode

n	3	4	5	6	7	8
Lmea(nH)	13.368	15.932	16.774	17.287	17.688	18.241
Lcal (nH)	12.066	14.027	15.493	16.277	16.645	17.038
Error	0.097	0.119	0.076	0.058	0.059	0.065

Supplementary Table 6 Summary of the pioneering works about transmission efficiency

**Power**	**Frequency**	**Gap (mm)**	**Transmitter area** (cm^2)	**Receiver area** (cm^2)	**Materials**	**Efficiency η/%**	**Efficiency with strain**	**References**
0.1 mW	20 MHz	2	~5.72	~4.55	Ag NFs	~40%	~15%	This work
--	3 ~ 5GHz	1	--	--	Ag NWs	--	--	*Nat. Commun.* **2017**, 14997
--	50MHz	5	--	~1.13	Ag NFs	21.5%	--	*Sci. Adv.* **2018**, eaap9841
--	13.4MHz	--	--	~1.77	Cu NPs	--	--	*Adv. Sci.* **2018**, 1801146
0.1 W	6.78 MHz	15	21	0.785	30 AWG wire	10 ~ 20%	--	*ISCAS*, **2007**, 2080-2083.
0.4 ~ 2 W	500 KHz	--	520	50	--	35%	--	*EPE*, ***2009***, 1-10
0.794 W	27 MHz	15	--	~4	--	80%	--	*ISABEL*, **2010**, 1-5
1.2 W	500 KHz	130	314	314	Wire windings	40%	--	*ECCE*, **2013**, 2239-2244
25.6 W	13.56 MHz	--	~28.3	~28.3	concentric coils	73.4 %	--	*IEEE MTT-S International*, **2012**, 1-3.
50 W	3.54 MHz	300	1257	1257	Cu on the PCB layouts	80%	--	*ECCE*, **2013**, 1917-1924
100 W	20 KHz	700	707	707	Litz wires AWG 36600	95.4%	--	*IEEE MTT-S International*, **2012**, 83-86.
2 KW	5 ~ 50 KHz	50 ~ 80	1385~3849	1385~3849	Litz wires	85%	--	*ECCE*, **2009**, 2081-2088
5 KW	20 KHz	246	21*103	21*103	AWG36 Litz wire	90%	--	*IEEE Trans. Ind. Inform.*, **2012**, 585-595

Supplementary Notes

Supplementary Note 1: Microstructure of Ag NFs under tensile strain

(a) SEM images of Ag NFs with different strains

The SEM images of Ag NFs electrodes with no stress are shown in the Supplementary Figure 2. The Ag NFs electrodes in the original state are randomly stacked and their surface is very smooth. The intrinsic gaps of Ag NFs show a good optical transparency of the electrode.

Fractures or cracks will generate on the Ag NFs electrodes with tensile strain increased. The morphologies of four samples under different strains are shown in the Supplementary Figure 3, which are at the same preparation processing conditions. These results show that the fracture of Ag NFs surface is rough and it has obvious difference from the intrinsic Ag NFs gap. On the other hand, the SEM images also depict that there are still effective conductive paths under tensile strain for the random orientation Ag NFs. These cracks will further expand with the increasing deformation, leading to the increment of the parasitic capacitance and the reduction of its inductance. In particular, the electrode loses its conductivity and will be completely transformed into a capacitor when the deformation exceeds the range that the Ag NFs can be withstand.

Moreover, the SEM in-situ observation are employed to further analysis the process of crack initiation and propagation by using an array pattern marker, as shown in the Supplementary Figure 4. It also indicates that the cracks expand with the increasing strain, in keeping with the previous discussed.

(b) Mechanical property of the PDMS substrate

The main agents and curing agents of the PDMS (SYLGARD184 Dow Corning) are mixed thoroughly in a weight ratio of 10:1 and then degassed for 5 min to remove air bubbles. Then, spinning it on the glass substrate and drying in the oven at 120 ℃ for 20 min. Supplementary Figure 5 shows the uniaxial tensile tests to evaluate the mechanical properties of the PDMS, which shows an ultimate stress of 2.238 MPa at

the strain of 144%. And it could be found that PDMS is in the elastic region within the 100% deformation which could satisfy the human body skin deformation.

(c) Conductivity and permeability of various materials

The magnetic permeability and electric conductivity of the materials are also important parameters for the device performance, including PDMS and metal nanofibers, as shown in Supplementary Table 2.

In this work, we also have fabricated a variety of metal nanofibers by using this method, including silver (Ag), gold (Au), platinum (Pt), copper (Cu), chromium (Cr), nickel (Ni). It could be noticed that this patterned preparation process is very suitable for Au NFs, Pt NFs and Ag NFs, Cu NFs, because these metals perform excellent ductility. However, the Cr NFs and Ni NFs will rupture on the surface of water, which make it impossible to form a hard film. Supplementary Figure 6 shows the patterned electrodes of Au NFs and Cu NFs. (Anderson, J. Malleability and Ductility of Metals. SCI AM, 1869, 21, 41-341)

(d) Contact angle of the Ag NFs film

The Ag NFs will attach to the PDMS substrate owing to the Van der Waal's force while drying, and the surface of the Ag NFs film will become hydrophobic when the Ag NFs are firmly bonded to the substrate. As shown in the Supplementary Figure 7, the contact angle of Ag NFs on the PDMS substrate via air gun blowing dry is 58.60°. After fully drying the Ag NFs at 100℃, it could be found that the contact angle becomes 127.73° which means the Ag NFs surface is hydrophobic, indicating that the water molecules in it have been completely evaporated and Ag NFs attach well to the PDMS substrate.

Supplementary Note 2: Analysis of Skin Effect of the Ag NFs electrode

The skin effect refers to be the phenomenon that an alternating electric current (AC) will be redistributed at the surface of conductor, and the current density is the maximum near the surface of the conductor. The electric current flows mainly in the "skin" of the conductor, between the outer surface and the level called the skin depth. The skin effect causes the increment of the conductor effective resistance at higher frequencies, because the skin depth will reduce the effective cross-section of the conductor.

The general formula for the skin depth is

$$\delta = \frac{1}{\sqrt{\pi f \mu_r \mu_0 \sigma}} \tag{2-1}$$

δ: skin depth in meters

f: frequency of the current in Hz

μ_r: relative magnetic permeability of the conductor

μ_0: permeability of free space, $4\pi \times 10^{-7}$ H/m

$\mu = \mu_r \times \mu_0$

σ: conductivity of the conductor

According to the literature, the conductivity of Ag is 5×10^7 S/m and its permeability is 1.2566×10^{-6} H/m. Substituting the conductivity and permeability of Ag into the formula, the skin depth is approximately 1 μm at alternating current of 5 GHz, and the skin depth is further increasing when the frequency decreases to the megahertz. More theoretical calculations of the skin depth of Ag at different frequencies are shown in Table 3. For our as-synthesized patterned Ag NF electrodes, the core-shell structure (Supplementary Figure 8) can weaken the resistance variation caused by the skin effect considerably. Meanwhile, the diameter of the Ag NFs is around 700 nm and several nanofibers are stacked together with a thickness of 2-3 μm., which is less than the skin depth overall. Therefore, the change in resistance owing to the skin depth could be negligible.

After the magnetron sputtering, a thin layer of Ag is coated on the surface of the

nanofibers to form the PVA/Ag core/shell structure. Moreover, the Ag shell is perfectly preserved after dissolving the PVA NFs on the water surface. The Supplementary Figure 8 shows the cross-sectional SEM images of the network structure of Ag NFs, illustrating the conformal coating and uniformity of the core/shell structure. The Ag NFs have extremely high aspect ratio and their attachment to the stretchable substrate (PDMS) leads to the accommodation of a high strain.

Supplementary Note 3: Theoretical calculation of Ag NFs electrodes inductance

(a) Theoretical analysis

The electrical performance of Ag NFs electrodes is mainly affected by the inductance and resistance. Resistance is an important parameter for electrode quality factor. From the perspective of electrode dimension, the electrode length or width will affect the resistance of electrode. On the other hand, NFs density or the sputtering duration also affect the resistance of electrode in terms of the material characteristic. Hence, it is essential to reduce the Ag NFs electrode resistance for the higher quality factor by improving the preparation process.

Inductance is another important parameter for the radio frequency characteristics of the Ag NFs coils, which is mainly affected by three aspects (length and width, shapes and coupling effect).

i) Straight electrodes with various length and width

According to Supplementary Figure 9a-b, the relationship of the straight electrode between the length or width and the inductance can be expressed:

$$L = \frac{\mu_0 l}{2\pi}[\ln(l/d) - 0.75] \tag{3-1}$$

μ_0: permeability of free space, $4\pi \times 10^{-7}$ H/m

l: length of straight electrode

d: width of straight electrode

ii) Single-turn electrodes with various shapes

For single-turn electrodes with different shapes, the theoretical calculation of inductance are shown below:

$$L = N - G + A - Q \tag{3-2}$$

N: parameter determined only by the shape and size of the wire axis;

G, A, Q: parameters determined by the shape and size of the wire cross section and the current distribution in the wire cross section. The A and Q could be ignored in this article due to the small amounts.

$$L = N - G \tag{3-3}$$

Regular polygon electrode inductance (*N*):

$$N = \frac{\mu_0 a n}{2\pi}[\ln a + f(n)] \quad (3\text{-}4)$$

n: Number of sides of the polygon

a: Length of one side

f(*n*): Parameter related to the number of sides of the polygon shown in Table 4

Circular electrode inductance (*N*):

$$N = \mu_0 R[\ln 8R - 2] \quad (3\text{-}5)$$

R: Radius of the circular electrode

$$G = \frac{\mu_0 l}{2\pi}[\ln(d/2) - 0.0584] \quad (3\text{-}6)$$

μ_0: permeability of free space, $4\pi \times 10^{-7}$ H/m

l: length of regular polygon electrode

d: width of regular polygon electrode

As shown in Supplementary Figure 10a-c, for the electrodes with the same length but different shapes, the circular electrode exhibits the highest inductance due to its larger area. Meanwhile, Supplementary Figure 10b shows that the greater of the electrode length have the smaller inductance change at the same area variation ratio. The results indicate that *L* is mainly affected by the dimension and layout of the coil, especially the electrode length. Supplementary Figure 10c illustrates that the inductance variation versus the circular electrode radii, which shows the same trend as the experimental results.

iii) Coupling effect of multi-turns coil electrodes

For the planar spiral coil electrode, the specific formula could be obtained by fitting as follows:

$$L = \frac{\mu_0}{8\pi}\omega^2 d\psi \quad (3\text{-}7)$$

μ_0: permeability of free space, $4\pi \times 10^{-7}$ H/m

ω: number of turns of the coil

d: average diameter of the coil

ψ: a specific parameters which was determined by the cross section of electrode

It could be found that the spiral coil inductance shows a square proportional relationship with turns, resulting from the double number of inductive coupling between the internal coil for each additional coil turn.

(b) Modeling analysis

As shown in Supplementary Figure 11, the quality factor is affected by the inductance and resistance. The resistance is strongly influenced by the dimension and material characteristics of the conducting electrodes, such as the length or width, NFs density and sputtering duration, and smaller resistance can be achieved by optimized synthesis process. Furthermore, we further analyze and fit the data to better control the Ag NFs electrode inductance (sheet resistance is 11.1 ~ 1.68 Ω/□, at >70% transmittance).

The straight Ag NFs electrodes are first investigated as the most basic structural unit, and the empirical formula of the straight electrode length and width relative to the inductance could be obtained:

$$L = \frac{\mu_0 l}{2\pi}[\ln(l/d) - 0.912] \tag{3-8}$$

It could be further calculated to obtain the following formula:

$$L_{straight} \approx 2 \times 10^{-7} \times l \times [\ln(l/d) - 0.912] \tag{3-9}$$

Then, the inductance of the coil reduces from a straight shape to a shapely pattern due to the existence of mutual inductance among different line segments. The inductance of curve electrode can be described as:

$$L = L_{straight} - \Delta L \tag{3-10}$$

For a certain length electrode, the circular electrode has the largest area. Hence, we use the circular electrode as a reference object to explore the inductance variation under different areas. The effect of area on the inductance could be obtained by derivation of the previous formula:

$$\Delta L = \frac{L_{circle}}{S_{circle}} \Delta S + A \tag{3-11}$$

As shown in Figure 1h, the inductance variation are linearly related to coil radius, and it could be fitted:

$$L_{circle} \approx 0.966L_{straight} - 1.867\times10^{-9} \tag{3-12}$$

$$\frac{L_{circle}}{S_{circle}} = \frac{19.095\times10^{-9}}{1.2732\times10^{-4}} = 14.998\times10^{-5} \tag{3-13}$$

Hence, the formula (3-10) can be modified as:

$$L{=}L_{straight} - \frac{L_{circle}}{S_{circle}}\Delta S - A = L_{straight} - 14.998\times10^{-5}\times\Delta S - A \tag{3-14}$$

After fitting and calculating the inductance of different shapes, empirical formula could be obtained:

$$L \approx L_{straight} - 1.4998\times10^{-4}\times\Delta S - 5.203\times10^{-10} \tag{3-15}$$

Where $L_{straight}$ = $2\times10^{-7}\times l\times[\ln(l/d)-0.912]$. Supplementary Table 5 shows the error between the theoretical and the measured value.

Supplementary Note 4: Inductance model and one-port scattering analysis

As shown in Supplementary Figure 12, one-port scattering analysis is used for the inductors. The scattering parameter S_{11} could be obtained which is equivalent to the reflection coefficient of the measurement port. In addition, the input impedance Z_{in} and impedance angle θ of the inductors under testing could be acquired[1].

$$S_{11} = \frac{b_1}{a_1} \tag{4-1}$$

$$Z_{in} = Z_0 \frac{1+S_{11}}{1-S_{11}} \tag{4-2}$$

$$\theta = \mathrm{arc}\frac{\mathrm{Im}[Z_{in}]}{\mathrm{Re}[Z_{in}]} \tag{4-3}$$

$$L = \frac{\mathrm{Im}[Z_{in}]}{\omega} \tag{4-4}$$

$$Q = \frac{\mathrm{Im}[Z_{in}]}{\mathrm{Re}[Z_{in}]} \tag{4-5}$$

where Z_0 = 50 Ω is the characteristic impedance of the measurement system. Z_{in} as a function of frequency shows the radio frequency characteristic of the inductors, where L and Q can be extracted from Z_{in} according to the above formula.

The lumped-parameter model of the inductor, consisting of inductance L, resistance R, and parasitic self-capacitance C, can be further simplified to the effective series resistance R_S and reactance X. The input impedance of the inductors can also be obtained by the series-parallel circuits theory, and the reactance X (imaginary part) of the circuit becomes zero at the self-resonance frequency f_0 for inductors.

$$Z_{in} = \frac{1}{\frac{1}{j\omega L + R} + j\omega C} \tag{4-6}$$

$$Z_{in} = \frac{R + j\omega L(1-\omega^2 LC) - j\omega R^2 C}{(1-\omega^2 LC)^2 + \omega^2 \mathrm{R}^2 \mathrm{C}^2} \tag{4-7}$$

$$\mathrm{Re}[Z_{in}] = R_S = \frac{R}{\left(1-\omega^2 LC\right)^2 + \omega^2 R^2 C^2} \tag{4-8}$$

$$\mathrm{Im}\left[Z_{in}\right] = X = \frac{j\omega L\left(1-\omega^2 LC\right) - j\omega R^2 C}{\left(1-\omega^2 LC\right)^2 + \omega^2 R^2 C^2} \tag{4-9}$$

$$f_0 = \frac{1}{2\pi\sqrt{LC}}\sqrt{1-\frac{R^2 C}{L}} \tag{4-10}$$

Inductance variation of the straight Ag NFs electrodes versus the tensile strain is shown in Supplementary Figure 13. Initially, denser NFs can be obtained with the increasing electrostatic spinning duration, resulting in poorer transparency and higher stretchability, and it is difficult to become a capacitor under tensile strain. Secondly, the inductance will increase at small extensional strains, which may be due to the variation of the electrode length. However, large cracks will appear on the electrodes when the strain exceeds the Ag NFs could withstand, and the electrode gradually loses its conductivity so that the inductance will continue to decline and become a capacitor.

Supplementary Figure 14 shows optical photographs and SEM images of the Ag NFs coil under four different electrospinning durations. It can be seen that the transparency of the Ag NFs coil gradually decreases with the denser Ag NFs density, resulting in higher conductivity and stretchability. Hence, we can infer that appropriate enhancement of the NFs density is an effective way to prevent the device from turning a inductor to capacitor under tensile strain. Moreover, Supplementary Figure 15 shows optical photographs of a circular electrode and three square electrodes under different tensile directions. The results show that the circular coil has more stable inductance and less resistance variation, indicating that the circular coil is more suitable for the wireless epidermal electronics. Supplementary Figure 16 shows the Smith chart of three-turn and four-turn spiral coils under tensile strain. The self-resonance frequency f_0 shifts toward lower frequencies owing to the increasing resistance.

Supplementary Note 5: Finite element simulation of the mechanical property

(a) Mechanical properties of nanofibers

Simulations of unidirectional, bidirectional and random orientation Ag NFs under tensile strain are carried out to exhibit the stress distribution by using a finite-element method (COMOSL) in three-dimensional space, as shown in Supplementary Figure 17a-c. It is worth noting that the Ag NFs have significant stress concentration when the deformation along the nanofiber's orientation, especially on the interconnection of the Ag NFs. Hence, unidirectional Ag NFs are susceptible to stress concentration which are not suitable for the stretchable devices.

For the bidirectional and random orientation Ag NFs with tensile strain, although the Ag NFs in one direction are in a state of high strain, Ag NFs in the other directions could maintain low stress which can stay intact to form a conductive path. The Supplementary Figure 17d shows the evolution of unidirectional Ag NFs under tensile strain. Upon stretching, the PDMS substrate with low effective stiffness presents elastic deformation to absorb stress and many fractures will generate on the Ag NFs along the tensile direction. Hence the Ag NFs electrode will change from an inductance to capacitance and the corresponding SEM (right) images illustrate the variation of the unidirectional Ag NFs under tensile strain.

The tensile properties of the bidirectional Ag NFs and more SEM images are shown in the Supplementary Figure 17e. It could be found that many fractures appear on the Ag NFs along the tensile direction, which is consistent with the simulation results. Nevertheless, Ag NFs along the other orientations are still kept intact and there exist various conductive paths in high strain. Moreover, random orientation Ag NFs have more orientations than that of bidirectional Ag NFs and it possesses more conductive paths under tensile strain. In addition, the SEM images also show the as-synthesized Ag NFs are naturally interconnected at their junctions. Hence, we speculate the random orientation Ag NFs perform the better tensile properties, which is an ideal material for epidermal electronics (even in the places with large strains of human body, including neck or joint, etc.).

(b) Mechanical properties of Ag NFs coils

Based on the above analysis, it could be found that Ag NFs with random orientation still exist various conductive paths even under high strain, so we equate the Ag NFs to a thin film to explore the optimal stress distribution under different shapes and design the better geometrical structure for stretchable devices. Detailed simulation results are shown below.

Simulations of stress distribution for different straight electrodes are shown in Supplementary Figure 18. We simplified the Ag NFs electrodes into a thin film in order to facilitate the calculation of the model. The Ag NFs electrodes with different lengths and widths are subjected to tensile analysis. The results show that the longer of the electrode length means intenser stress concentration in the middle of the electrode, and the Ag NFs is prone to fracture. However, for the Ag NFs electrode with different widths, the wider electrode means less stress distribution in the middle. To quantitatively analyze the stress variation of Ag NFs electrode, we select the middle point for stress calculation, the results show that the stress have increased from 0.176 MPa to 0.367 MPa with the increase of the length, but declined from 0.263 MPa to 0.259 MPa with the increase of the width.

For the Ag NFs electrodes with different shapes (including triangle, square, pentagon, hexagon, octagon and circular), the simulation results are similar to that of the nanofibers. The polygon electrode edges with the same orientation as deformation are easier to bear the larger stress which means the Ag NFs electrode are more likely to break. Moreover, the results also exhibit that the stress is easily to aggregate in the corner of the polygon electrode, which means the Ag NFs electrodes here are prone to fracture. Hence, it is better to choose the circular electrode as the stretchable devices so as to avoid the stress concentration at the corners.

Furthermore, we compared the stress distribution of circular electrodes with different lengths and widths. For the circular electrode, the electrode length does not affect the stress distribution excessively, but the wider electrode means the smaller stress at the boundary, which is more conducive to prepare stretchable devices. Therefore, the electrode width could be appropriately increased to obtain better tensile

properties when designing the circular electrode.

Finally, we simulated the stress distribution of the Ag NFs electrodes (including quadrangle and circular) with different tensile directions. The schematic diagram of COMSOL model is shown in Supplementary Figure 21a, and the Ag NFs electrodes are encapsulated with PDMS and the bottom end of the PDMS is fixed. Subsequently, stress is applied at the two upper corners of the PDMS substrate, and the results in Supplementary Figure 21b shows that the stress distribution of square electrodes is obviously greater than the circular electrodes, especially at the corners.

Supplementary Note 6: Transmission model and two-port scattering analysis

Two-port microwave scattering measurements are performed to characterize the wireless power transfer efficiency η, where two scattering parameters S_{11} and S_{21} are measured (Supplementary Figure 22)[2-5]. The power transfer efficiency is evaluated according to the following formula:

$$\eta = \frac{P_t}{P_{in} - P_r} = \frac{\left|S_{21}\right|^2 / Z_0}{\mathrm{Re}\left[\left|S_{11} + 1\right|^2 / Z_{in}\right]} = \frac{\left|S_{21}\right|^2}{1 - \left|S_{11}\right|^2} \tag{6-1}$$

The corresponding circuit model of the simple magnetic induction mode is shown below, which is equivalent to a T-type circuit. Based on the circuit theory of Kirchhoff's voltage law, the two-port S_{21} parameter can be deduced as follows:

$$\begin{pmatrix} V_G \\ 0 \end{pmatrix} = \begin{pmatrix} Z_0 + Z_{11} & j\omega M \\ j\omega M & Z_0 + Z_{22} \end{pmatrix} \begin{pmatrix} I_1 \\ I_2 \end{pmatrix} \tag{6-2}$$

$$Z_{11} = j\omega L_1 + R_1 \tag{6-3}$$

$$Z_{22} = j\omega L_1 + R_2 \tag{6-4}$$

$$V_2 = j\omega M I_1 + Z_{22} I_2 \tag{6-5}$$

$$S_{21} = \frac{b_2}{a_1}\Big|_{a_2=0} = \frac{2V_2}{V_G} = \frac{2j\omega M Z_0}{\left(R_1 + j\omega ML_1 + Z_0\right)\left(R_2 + j\omega ML_2 + Z_L\right) + (\omega M)^2} \tag{6-6}$$

Meanwhile, the S_{11} parameter can also be calculated via the reflection coefficient:

$$S_{11} = \frac{Z_{in} - Z_0}{Z_{in} + Z_0} \tag{6-7}$$

$$Z_{in} = R_1 + j\omega ML_1 + \frac{\left(\omega M\right)^2}{R_2 + j\omega ML_2 + Z_L} \tag{6-8}$$

$$S_{11} = \frac{\left[\left(R_1 - Z_0\right)\left(R_2 + Z_0\right) - \omega^2 L_1 L_2 + \left(\omega M\right)^2\right] + j\left[\omega L_1\left(R_2 + Z_0\right) + \omega L_2\left(R_1 - Z_0\right)\right]}{\left[\left(R_1 + Z_0\right)\left(R_2 + Z_0\right) - \omega^2 L_1 L_2 + \left(\omega M\right)^2\right] + j\left[\omega L_1\left(R_2 + Z_0\right) + \omega L_2\left(R_1 + Z_0\right)\right]} \tag{6-9}$$

Substitute S_{11} and S_{21} into the efficiency formula:

$$\eta = \frac{\omega^2 M^2 Z_0^2}{\omega^2 L_2^2 R_1 Z_0 + \omega^2 M^2 Z_0\left(R_2 + Z_0\right) + Z_0 R_1\left(R_2 + Z_0\right)^2} \tag{6-10}$$

where $Z_0 = Z_L = 50\ \Omega$ are the characteristic impedance and load impedance of the

measurement system, respectively[6]. The variables in the above-mentioned formula include the resistance R (R_1, R_2), inductance L (L_1, L_2, M), and frequency f. To better understand the effect of the above-mentioned parameters on the transfer efficiency, we use the variable control approach to research its effect on the efficiency according to the formulas (Supplementary Equations 6-2 to 6-10) and we assume that the parameters do not change with the frequency. Supplementary Figure 23 shows the effect of frequency (f) on the S parameters and the transfer efficiency while the frequency rises from 10 Hz to 100 MHz. It can be seen that S_{11} gradually increases with the frequency, indicating that most of the energy is reflected owing to the impedance mismatch. S_{21} reaches an extreme value at around 4 MHz which is the maximum power transmission frequency for the system. And the wireless transfer efficiency increases with the frequency and then becomes stable. Moreover, the results show that the transfer efficiency increases with the mutual inductance when M augments from 0.1 μH to 1 μH, and it decreases when R_2 rises from 10 Ω to 100 Ω.

$$\begin{pmatrix} V_G \\ 0 \end{pmatrix} = \begin{pmatrix} Z_0 + Z_{11} & j\omega M \\ j\omega M & Z_0 + Z_{22} \end{pmatrix} \begin{pmatrix} I_1 \\ I_2 \end{pmatrix} \tag{6-11}$$

$$Z_{11} = \frac{1}{\frac{1}{j\omega L_1 + R_1} + j\omega C_1} \tag{6-12}$$

$$Z_{22} = j\omega L_1 + R_2 \tag{6-13}$$

$$S_{21} = \frac{2V_2}{V_G} = \frac{2Z_{21}Z_0}{(Z_{11}+Z_0)(Z_{22}+Z_0) - Z_{21}Z_{21}}$$
$$= \frac{2j\omega M Z_0\left[\left(1-\omega^2 L_1 C_1\right) + j\omega C_1 R_1\right]}{\left[R_1 + Z_0\left(1-\omega^2 L_1 C_1\right)\right]\left(R_2 + Z_0\right) - \omega L_2\left(\omega L_1 + \omega C_1 R_1 Z_0\right) + \left(\omega M\right)^2\left(1-\omega^2 L_1 C_1\right) + j\left\{\omega L_2\left[R_1 + Z_0\left(1-\omega^2 L_1 C_1\right)\right] + \left(R_2 + Z_0\right)\left(\omega L_1 + \omega C_1 R_1 Z_0\right) + \omega^3 M^2 C_1 R_1\right\}}$$

(6-14)

$$S_{11} = \frac{(Z_{11} - Z_0)(Z_{22}+Z_0) - Z_{21}Z_{21}}{(Z_{11}+Z_0)(Z_{22}+Z_0) - Z_{21}Z_{21}}$$
$$= \frac{\left[R_1 - Z_0\left(1-\omega^2 L_1 C_1\right)\right]\left(R_2 + Z_0\right) - \omega L_2\left(\omega L_1 - \omega C_1 R_1 Z_0\right) + \left(\omega M\right)^2\left(1-\omega^2 L_1 C_1\right) + j\left\{\omega L_2\left[R_1 - Z_0\left(1-\omega^2 L_1 C_1\right)\right] + \left(R_2 + Z_0\right)\left(\omega L_1 - \omega C_1 R_1 Z_0\right) + \omega^3 M^2 C_1 R_1\right\}}{\left[R_1 + Z_0\left(1-\omega^2 L_1 C_1\right)\right]\left(R_2 + Z_0\right) - \omega L_2\left(\omega L_1 + \omega C_1 R_1 Z_0\right) + \left(\omega M\right)^2\left(1-\omega^2 L_1 C_1\right) + j\left\{\omega L_2\left[R_1 + Z_0\left(1-\omega^2 L_1 C_1\right)\right] + \left(R_2 + Z_0\right)\left(\omega L_1 + \omega C_1 R_1 Z_0\right) + \omega^3 M^2 C_1 R_1\right\}}$$

(6-15)

The primary coil usually has parasitic capacitance, so it is necessary to add the parasitic capacitance into the above presented wireless transmission model. The S parameter and the transfer efficiency can be deduced via two-port network theory and the circuit theory of Kirchhoff's voltage law (Supplementary Equations 6-11 to 6-15). It can be seen that the efficiency is the lowest when the primary coil resonates, so we

should focus on the transfer efficiency within the resonance frequency (Supplementary Figure 24).

The resonance frequency of the primary coil is 38.3 MHz, at which it has the maximum impedance and the impedance angle of zero (Supplementary Figure 25a). Supplementary Figure 25b shows the S_{11} parameter, impedance, and impedance angle of the stretchable spiral coils (1, 2, 3, 4, and 5 turns) versus the frequency. It can be seen that the self-resonance frequency f_0 shifts rapidly to lower frequencies with the spiral coil turns increasing, which is caused by the enhancement of the inductance. Furthermore, S_{11} gradually increases while S_{21} decreases with the variation of the lateral distances and rotation angles, indicating that the energy transmitted forward is reducing and more energy is reflected. The above-mentioned analysis implies that the coupling between two coils is significantly weakened when the relative position of the coil changes, resulting in the decline of the transfer efficiency. In addition, when the stretchable spiral coil is under tensile strain, the S_{11} parameter shows no distinct variation while S_{21} gradually decreases, resulting in a decrease in the transfer efficiency (Supplementary Figure 26). This is consistent with the theoretical calculation presented above.

Supplementary Note 7: Applications in functional wireless electronics

(a) Stretchable antenna for power transfer

The wireless power transfer efficiency is also an important parameter for the magnetic induction antenna. It has been reported that the transmission efficiency can reach 95%, and there is no obvious advantage from the perspective of power transfer efficiency for our devices and systems. However, few reports are available about stretchable antenna for power transfer with high transmission efficiency. In this work, the radio frequency characteristics of Ag NFs antenna is explored by designing the optimal geometry for better performance. The results show that a five-turn spiral coil contains 40% power transfer efficiency at 20 MHz, and the transfer efficiency was approximately 15% even under the strain of 100% based on the magnetic inductive coupling mode. Supplementary Table 6 shows the comparison of the current magnetic inductive transfer efficiency with this work, indicating that Ag NFs antenna has broad application in human epidermal electronics.

The specific dimensions of the chip LED used in this work are shown in the Supplementary Figure 27. The chip LED could achieve a good contact with Ag NFs spiral coil by using lithography and 3D printing technology. It will be lighted using the external coil coupling with the Ag NFs spiral coil, indicating that the Ag NFs could be combined with traditional small electronic devices to achieve more human-machine interaction.

(b) Battery-free NFC devices & audio signal detection

$$L=\frac{1}{\left(2\pi f\right)^{2}C}=\frac{1}{(2\pi\times 13.56\times 10^{6})^{2}\times 50\times 10^{-12}}=2.7\times 10^{-6} \tag{7-1}$$

The Ag NFs spiral coil tags can operate in a battery-free mode via an external reader (i.e., any NFC-enabled smartphone, tablet, or watch) for data and power transmission by magnetic inductive coupling using near-field communication (NFC) protocols. This chip (NTAG 213) is based on the ISO/IEC 14443 Type A standard, with 144 bytes of user-programmable read/write memory and input capacitance is 50 pF. Therefore, it is essential to fabricate the spiral coil antenna with a specific inductance at the operating frequency. Supplementary Figure 28 shows that the Ag

NFs spiral coil antenna has an inductance of 2.7 μH and a resistance of 232 Ω at 13.56 MHz. The ability of the stretchable coil to receive information over a long distance was verified. Frequency modulation (FM) refers to the encoding of information in a carrier wave by varying the instantaneous frequency of the wave. The frequency modulation module RC-127 is used for modulating audio signals, and a spectrum analyzer (N9030B Keysight) is used to detect the signals wirelessly. Supplementary Figure 29 shows the auditory spectrograms of 5 males and 5 females, which indicates that different letters have different frequency distributions and the female spectrum is slightly higher than the male spectrum.

(c) Ag NFs antenna gain radiation pattern

The carrier frequency used in this work is 87 MHz to 108 MHz, and we choose 100 MHz as carrier wave to transmit audio information. In order to deeply understand the RF performance of Ag NFs antenna, we simulated the gain of the antenna via finite-element method (COMSOL) in three-dimensional space. The reference model is simulated with the same dimension as that of Ag NFs antenna and it is enclosed by a numerical version of a sphere which is a perfect matched layer (PML). The Ag NFs antenna is encapsulated with PDMS, and the lumped port has 50 Ω for reference impedance and all domains is filled with air. Supplementary Figure 30a shows that the Ag NFs spiral coil antenna gain at 0.1 GHz, and it could be seen that the antenna's gain is -15.48 dBi. Subsequently, the most important tag performance is the read range - the maximum distance at which RFID reader can detect the backscattered signal from the tag. Therefore, we further analyzed the antenna's gain at 0.9 GHz which is the UHF bands for RFID systems, and it could be seen that the antenna's gain is -0.16 dBi as shown in the Supplementary Figure 30b. According to the literature[7], under the hypothesis of polarization matching between the reader and tag antennas, the maximum activation distance of the tag along the (θ, ϕ) direction is then given by Supplementary Equation 7-2.

$$d_{\max}\left(\theta,\phi\right)=\frac{c}{4\pi f}\sqrt{\frac{EIRP}{P_{chip}}\tau G_{tag}(\theta,\phi)} \tag{7-2}$$

c: velocity of the light in free space;

f: working frequency of the antenna;

P_{chip}: sensitivity of the chip;

G_{tag}: gain of the antenna;

EIRP: the effective power transmitted by the reader;

The factor

$$\tau = \frac{4R_{chip}R_A}{\left|Z_{chip} + Z_A\right|^2} \qquad (7\text{-}3)$$

is the power transmission coefficient, which accounts for the impedance mismatch between the antenna ($Z_A = R_A + jX_A$) and the microchip ($Z_{chip} = R_{chip} + jX_{chip}$). The impedance of microchip depends on the input power, and its input reactance is strongly capacitive since the transponder includes an energy storage. The antenna impedance should be inductive in order to achieve conjugate matching, and a large impedance phase angle needs to be obtained. Beyond $d_{\max}$, the power collected by the tag decreases below the microchip sensitivity, and the tag becomes unreachable. The input impedance and sensitivity of the chip is Z_{chip} = 12 - j150 and 12μW, the impedance of prepared Ag NFs antenna could be Z_A = 98 + j150 to achieve the impedance matching. Hence the power transmission coefficient could be calculated as 0.0433, and substitute τ into the Supplementary equation 7-2 to estimate the maximum reading distance with a 4 W EIRP condition.

$$d = \frac{3\times10^8}{4\pi\times9\times10^8}\sqrt{\frac{4}{12\times10^{-6}}\times0.0433\times0.96} = 3.12m \qquad (7\text{-}4)$$

In addition, we also analyzed the Ag NFs antenna’s gain variation under tensile strain and it could be noticed that the gain decreases from -0.16 dBi to -0.33 dBi. The maximum reading distance with a 4 W condition changes to the 3.05 m, which scarcely change compared to the original device, indicating that the Ag NFs antenna has extensive applications in wearable electronic devices.

Supplementary Methods

Preparation process of the Ag NFs electrode

The procedure to prepare the patterned stretchable transparent electrode is described as below:

1. Electrospinning: Polyvinyl alcohol water solution (10%) is loaded into a 10mL plastic syringe with a blunted G7 needle for electrospinning. Air humidity is maintained at around 25% and the flow rate of solution is 0.4 ml/h. The negative voltage of -4 kV is applied on the needle, and the positive voltage of 9 kV on the circular substrate (acrylic frame with aluminum foil). The distance between the tip of needle and substrate is 10 cm.

2. Metallization: The PVA NFs are then coated with a thin layer of silver by magnetron sputtering (PVD75 Kurt J. Lesker, Ar, 4 mTorr, 100 W, 15 min) to form a core-shell structure Ag NFs. The average diameter of Ag NFs is about 600 nm.

3. NFs transfer process: Firstly, PDMS is prepared at a ratio of 10: 1 and stirred to make it homogeneous. And then PDMS is spin-coated on a glass substrate. After that, the substrate with PDMS is placed on the vacuum oven to remove the bubbles at the pressure of -0.1 MPa. Subsequently, the substrate will be dried in the oven for 20 min at 120 ℃. Next, the fresh-obtained Ag NFs is put on the surface of water, and then transfer it on the PDMS substrate to ensure a flat Ag NFs on the substrate without wrinkles. The Ag NFs will form a stronger adhension with PDMS substrate after drying.

4. Photolithography: The negative photoresist is spin-coated on the surface of the Ag NFs. After exposure and development, the remaining photoresist as a mask layer would protect the Ag NFs from chemical etching.

5. Etching: The uncovered Ag NFs will be etched by the 5 M dilute nitrate solution in 30 second. Use acetone to clean the sample several times to remove the residual photoresist. The remaining Ag NFs is the desired patterned electrode.

6. Packaging: Another PDMS layer is spin-coated on the device to protect electrode.

Fabrication process of the integrated circuit

The whole preparation process of the integrated circuit is summarized as below:

1. The PDMS is spin-coated on a glass substrate as the elastic substrate.

2. The first Ag NFs electrode layer is fabricated on the PDMS substrate, which is treated as the bottom electrode (details can be found in Part I).

3. Then, a PDMS insulation layer is covered on the bottom electrode using a plastic syringe to avoid short circuit between coil and bottom electrodes.

4. The second Ag NFs electrodes is transferred on the device to fabricate the coil electrodes.

5. Spin-coating another PDMS film on the device as the encapsulation layer to protect electrode.

6. Finally, peel off the whole device from the glass.

Different patterns used in the experiments

Draw different patterns through software and convert them to 'cif' files, and the corresponding masks could be obtained by using the direct write systems (Heidelberg Instruments DWL 66+). Several patterns used in the experiments were fabricated, including the office logo, a pair of cartoon figure, plant leaves and various spiral coils. The high-precision patterned Ag NFs electrode will be fabricated with the different masks via photolithography and wet etching.

Schematic photographs of these steps are shown in Supplementary Figure 1.

Finite element method (FEM) modelling.

The stretching mode is calculated by using the Solid mechanics modulus in COMSOL Multiphysics, and this model involves in the Ag NFs electrodes covered with PDMS. The PDMS substrate is fixed at one end while applying displacement on the other end. The Von Mises Stress yield criterion is applied to observe the stress distribution of Ag NFs. The wireless power transfer mode is calculated by using the radio frequency modulus. The mode consists of two Ag NFs coil encapsulated with PDMS, which is settled in a air domain with perfectly matched layers (PML). The surrounding PMLs are necessary to absorb the radiation from the transmitting antenna and analyze the antenna coupling in the infinite free space. A lumped port with 50 Ω reference impedance is assigned to excite or terminate the antennas.

Supplementary References

1. Wojda, R. P. & Kazimierczuk, M. K. Winding resistance of litz-wire and multi-strand inductors. *IET Power Electron*. **5**, 257-268 (2012).
2. Sample, A. P., Meyer, D. A. & Smith, J. R. Analysis, Experimental Results, and Range Adaptation of Magnetically Coupled Resonators for Wireless Power Transfer. *IEEE Trans. Ind. Electron*. **58**, 544-554 (2011).
3. Boys, J. T., Elliott, G. A. J. & Covic, G. A. An appropriate magnetic coupling co-efficient for the design and comparison of ICPT pickups. *IEEE Trans. Power Electron*. **22**, 333-335 (2007).
4. Wang, M., Shi, Y. Y., Gao, W. K., Shen, M. H. & Shen, M. S. Design and optimization of hybrid resonant loops for efficient wireless power transfer. *Int. J. Circuit Theory Appl*. **46**, 328-342 (2018).
5. Enomoto, A. et al. Wireless implantable coil with parametric amplification for in vivo electron paramagnetic resonance oximetric applications. *Magn. Reson. Med*. **80**, 2288-2298 (2018).
6. D. M. Pozar, Microwave Engineering, Wiley, Hoboken, NJ, USA 2011.
7. Marrocco, G. The art of UHF RFID antenna design: Impedance-matching and size-reduction techniques. *IEEE Antenn. Propag. M* **50**, 66-79 (2008).
