# Supplementary material for: High precision epidermal radio frequency antenna via nanofiber network for wireless stretchable multifunction electronics
Source: Nat Commun. 2020 Nov 6;11:5629. doi: 10.1038/s41467-020-19367-8 (PMC7648760; doi:10.1038/s41467-020-19367-8)
Supplement: Supplementary file 3 — Description of Additional Supplementary Files [file 41467_2020_19367_MOESM3_ESM.pdf]

Description of Additional Supplementary Files

File name: Supplementary Movie 1
Description: Wireless power transmission characteristics of the coils under tensile strains

File name: Supplementary Movie 2
Description: Long-distance communication of the coils under tensile strains
